# Authors’ response: Bias in the vaccine effectiveness estimates of one-dose post-exposure prophylaxis against mpox

**DOI:** 10.2807/1560-7917.ES.2023.28.34.2300442

**Published:** 2023-08-24

**Authors:** Laura Montero Morales, José Francisco Barbas del Buey, Marcos Alonso García, Jesús Iñigo Martínez, Noelia Cenamor Largo, Susana Jiménez Bueno, Araceli Arce Arnáez

**Affiliations:** 1 Directorate General of Public Health, Regional Ministry of Health of Madrid, Madrid, Spain

**Keywords:** Spain, sexually transmitted infections, men who have sex with men - MSM, outbreaks, post-exposure prophylaxis, vaccines and immunisation, epidemiology, statistics

**To the editor: **We appreciate the readers’ letter, their interest and the comments made to our article ‘Post-exposure vaccine effectiveness and contact management in the mpox outbreak, Madrid, Spain, May to August 2022’ [1], to which we respond below.

The justification for the use of vaccination as post-exposure prophylaxis (PEP) in mpox (formerly monkeypox) is based on the need to offer protection to the people at greatest risk of becoming ill, who are the contacts of patients already infected. In accordance with this reasoning, in May 2022 the World Health Organization (WHO) indicated the possibility of vaccinating contacts [2] and included, from the moment of the declaration of the outbreak as a public health emergency of international concern (PHEIC) [3], the recommendations of vaccination as PEP and pre-exposure prophylaxis (PrEP). Regarding PEP, the recommendation was to ‘Consider the targeted use of second- or third-generation smallpox or monkeypox vaccines for post-exposure prophylaxis in contacts, including household, sexual and other contacts of community cases and health workers where there may have been a breach of personal protective equipment (PPE)’.

In Europe, the European Centre for Disease Prevention and Control (ECDC) established in July 2022 [4] that ‘Modelling the efficient use of vaccines indicates that PrEP vaccination would be the most efficient strategy when there is less effective tracing. The modelling also suggests that post-exposure prophylaxis (PEP) vaccination of contacts would offer a marginally more efficient approach if there are both higher uptake levels and more effective tracing (as fewer vaccines would be needed for a relatively larger increase in the probability of outbreak control per vaccinated individual), while the absolute probability of outbreak control with PEP vaccination is still lower than with PrEP vaccination. In settings where higher vaccine uptake is expected, PEP vaccination of close contacts of cases should also be considered, or even ring vaccination. Among these, contacts with a high risk of developing severe disease, like children, pregnant women, and immunocompromised individuals, should be prioritised’. By October 2022 [5], the ECDC still considered that ‘Mpox vaccines can be used as post-exposure vaccination (PEPV) or primary preventive (pre-exposure) vaccination (PPV)’.

Therefore, in this outbreak, the recommendations for the use of PEP have been maintained, regardless of the length of the incubation period and despite the fact that studies on vaccine effectiveness in this modality are very scarce.

We agree with the authors of the letter regarding the short duration of the window of opportunity for PEP vaccination against mpox, as expressed in the discussion of the article: ‘our main challenges were the identification and vaccination of contacts within the first 4 days after exposure. From the moment the index case sought medical care, the time elapsed between confirmation of the diagnosis, notification of the case, conduction of the epidemiological survey, communication with contacts, assessment of risk assessment to offer PEP and appointment exceeds 4 days in a high percentage of the cases. This makes it difficult to achieve the objective of vaccinating close contacts within 4 days in order to prevent the infection’ [1]. It should be considered that the short incubation periods could be the result of co-primary cases who engaged in group sexual practices and the extensive difficulties of contact tracing.

Regarding the timing of vaccination in relation to the incubation period, it should be taken into account that ‘The effectiveness of postexposure vaccination varies widely depending on disease course, in terms of individual immune response and population-level spread. […] postexposure vaccination modifies or prevents clinical disease among those who are already infected.[…] Measurable benefits may occur if the vaccine stimulates an immune response faster or greater than that provoked by the natural infection alone. For smallpox, the vaccine induces antibody response 4–8 days before the variola virus does, […]’ [6], which would justify the possible interference of the vaccine with infection despite the delay in vaccination. In our study, comparing vaccinated and unvaccinated patients, we obtained an adjusted VE of 71.6% (95% CI: 18.1–90.2) for the reduction of general symptoms and 85.5% (95% CI: 26.7–97.1) for the reduction of polysymptomatic disease. These findings would make it plausible that, for the smallpox vaccine, the ‘revaccination of previously vaccinated individuals at any time during the first week of the incubation period was largely protective, and that revaccination done even as late as the second week of the incubation period attenuated disease and prevented most deaths’ [7].

As mentioned in the letter to the editor, there are contacts in the study who were not offered PEP. The main reason for this was the shortage of vaccines in the initial phase of the outbreak, which meant that vaccines had to be requested from the Spanish Ministry of Health with an individualised and exhaustive justification for each contact for whom the vaccine was requested. Therefore, the vaccination capacity during the first month after the implementation of PEP was of about six persons per day. As a reference, by 1 July 2022, 436 cases of mpox had already been reported in the Madrid region, so it was not possible to offer the vaccine to all close contacts. Because of this, vaccination priority was established according to the nationally agreed contact prioritisation criteria [8,9], prioritising close contacts at high risk of disease or high risk of exposure: immunocompromised persons, pregnant women, children of any age and healthcare or laboratory personnel affected by accidental exposure. If vaccines were still available, these were then offered to contacts at high risk of exposure: sexual partners of household members/cohabitants with no possibility of self-isolation.

It should be noted that in our study we have taken into account the possible incidence bias as indicated in the discussion. ‘Another potential limitation would be the possible existence of an incidence bias that would cause contacts with early disease onset to be in the unvaccinated cohort because they failed to arrive in time for vaccination, which would lead to an overestimation of VE. To assess the possible magnitude of such an incidence bias, an intention-to-treat sensitivity analysis was performed, which showed little change in VE (85.4% for intention-to-treat vs 88.8% for estimated VE)’ [1]. This shows that the importance of the possible bias is negligible.

The authors of the letter also suggests a possible immortal time bias in the data analysis. Firstly, it should be noted that the follow-up ‘given the relatively short duration of this study, which was sufficient to detect new incident cases based on the incubation period described for the disease and short enough to avoid repeated risk exposures outside the cohabitation setting’ [1] has been short and the importance of immortal time bias ‘depend(s) directly on the amount of total person-time misclassified or excluded. Thus, the longer the exposure window, the more immortal time is misclassified, the larger the bias. Alternatively, if the exposure window is short, the impact of the bias will be negligible’ [10,11]. This bias has been described mostly in observational studies of chronic diseases with long exposure and follow-up time windows [11] or with life-long conditions [12].

The immortal time bias does not apply to an intention-to-treat analysis even if treatment or exposure begins after study entry. Participants included in the treatment or exposure group are analysed as treated or exposed even if the event occurs before the planned treatment or the start of the treatment or the exposure is delayed from the time of study entry, since all participants are analysed as if they had received treatment at the start of follow-up or study entry [13]. This means, participants included in the intention-to-treat analysis who are not vaccinated are counted as vaccinated even if they are not. Therefore, with an intention-to-treat analysis, the VE estimate is the maximum possible deviation because in unvaccinated participants included in intention-to-treat the effect of vaccination is null.

Taking into account the suggestions by the authors of the letter, if an intention-to-treat analysis is performed including as vaccinated those patients who were not vaccinated because they had symptoms before contacting them or before the vaccination date, as well as those who refused vaccination, an adjusted VE of 74.6% (95% CI: 55.4–85.5) is obtained. If the same analysis is performed, without taking into account follow-up time, using logistic regression adjusted for the same factors, the adjusted VE is 78% (95% CI: 56.9–88.8).

The authors of the letter state that an increasing VE should be obtained with delayed vaccination because there would have been a significant and sustained increase in immortal person-time in each of the groups, which, as indicated above, does not apply in the intention-to-treat analysis. However, as can be seen from the results by time from exposure to vaccination, this does not occur.

The authors of the letter indicate that, in order to avoid immortal time bias, we should have performed an analysis ‘including vaccination in the model as a time-varying exposure’. The introduction of the variable implies the definition of a deterministic transfer function of the effect of vaccination, dependent on the passage of time (since at day 0 its influence must be zero). This leads, among other problems, to arbitrariness in the choice of the transfer function, depending on the elements known about the vaccine response (largely unknown at present), and on the importance given to them by the research team. In addition, it would not take into account that a live attenuated virus vaccine could interfere with a secondary infection by the same or closely related virus [14-18] through the superinfection exclusion (SIE) mechanism. The SIE is described as ‘the ability of an established virus infection to interfere with a secondary infection by the same or a closely related virus, has been described for different viruses, including important pathogens of humans, animals, and plants’ [19-21]. ‘These interactions, so far as discovered, are antagonistic in nature^’^ [22,23]. Special consideration should be given to this effect in cohabitants or repeated sexual contacts who may have further exposures after the onset of symptoms of the index case and who have been the group prioritised for vaccination due to vaccine shortages.

Consequently, the implementation of vaccination as a time-varying exposure should include the possibility of SIE within the model as a stochastic component of which we do not know the probability, which together with the choice of the transfer function would increase the possibility of manipulation of the data by the researchers. For this reason, in our design we opted for an analysis closer to the ideal of experimentation (intention-to-treat analysis). Even so, we have performed an analysis following the ‘Landmark approach’ [13], eliminating both the cases who ended their follow-up before the seventh day (21 participants of whom 20 are cases and one lost to follow-up) as well as this initial follow-up time (immortal time) from all those who remained and obtained an adjusted VE of 82.6% (95% CI: 61.1–92.2) which does not vary much from the VE in the intention-to-treat analysis of our study 85.4% (95% CI: 72.6–92.2).

In summary, given the intention-to-treat analysis, the external validity of the analysis would lack the problems indicated in the letter, as the data would be extrapolated to the high-risk and high-exposure population in which PEP was indicated in real world conditions.
